# Promoting Public Health Through Drone Sports Within Diverse Communities of Middle- and High School Students

**DOI:** 10.3389/phrs.2024.1608117

**Published:** 2024-12-17

**Authors:** Ruby H. N. Nguyen, Isabel J. Ricke, Madelyn G. Allen, Martin C. Wetherall

**Affiliations:** ^1^ Division of Epidemiology and Community Health, University of Minnesota School of Public Health, Minneapolis, MN, United States; ^2^ RdyTechGo, Minneapolis, MN, United States

**Keywords:** public health, engagement, outreach, K-12 education, workforce development

## Introduction

A long-standing challenge to sustaining a robust public health workforce has been ineffective recruitment of young minds. We implemented a successful middle- and high school student engagement program that married an introduction to public health to drone sports to create an interactive and age-appropriate experience.

Over the last decade or two, undergraduate public health programs have been established to introduce public health curriculum to students earlier in their education [1], and for bachelor’s degree-holding public health practitioners to be prepared to enter the field immediately after graduation.

Data have shown that the undergraduate programs are racially and ethnically diverse; thus, these graduates better reflect the communities that public health serves. Using data from the National Center for Education Statistics, in 2020, 17% of undergraduate public health degree graduates were Hispanic/Latino, 15% were Black/African American, 13% were Asian, 4% were multiracial, and 1% were American Indian/Alaska Native; 45% of bachelor’s degree holders in public health were White [2]. However, such sustained successes of undergraduate programs rely partly on early introduction to public health before college while students are in middle- or high school.

Outreach to pre-college students (kindergarten through 12th grade in the U.S., K-12) has long been adopted by major Science, Technology, Engineering and Math (STEM) organizations to inspire young students to enter STEM professions, and thus advance the future workforce [3]. There are numerous established partnerships between professionals and K-12 STEM education that allow for an introduction to the profession. These introductions are often through fun and exciting hands-on experiences such as exploding chemistry experiments, electrifying physics exhibits, laboratory tours of brains for neuroscience, or programming robots and other autonomous machines. However, public health has traditionally not been considered a STEM field and, therefore, is not included in such pathway programs for young students.

Using the engagement principles of existing STEM outreach and engagement programs, we sought to develop an inspiring, interactive STEM-based program for a racially diverse group of middle- and high school students that would allow us to promote public health as a field, a career option for the students, and to address community health needs. Here, we describe our successful pilot program for middle and high school students, which merged an introduction to public health with interactive drone piloting and provided a pragmatic approach to the intersection and application of STEM activities with public health.

## Identification of the Local Needs for the Future of Public Health

Health indicators for the state of Minnesota, where this program was based, are strong and, in many respects, lead the nation. However, racial/ethnic disparities in this state are some of the most stark in the nation. For example, the state’s infant mortality rate is less than the average for the nation (4.8 versus 5.4 infant deaths per 1,000 births) [4]. Yet, the American Indian/Alaska Native infant mortality rate is 2.1x higher than the state average, and that for African American/Black is 1.7x than the state average [4]. Societal determinants and behaviors, and not the construct of race, drive these disparities [5]. Therefore, our public health workforce ought to be diverse in order to adequately address such needs from diverse communities. To meet this need, we focused on community outreach, engagement, and partnerships with underserved and under-resourced communities in the metropolitan Minneapolis/St. Paul area of Minnesota.

## Community Collaboration

Our school of public health resides in a university that serves a land-grant mission, which is to provide resources, education, and opportunities to the communities of the state. As part of our mission, we provide pre-college activities (such as camps) to youth. These opportunities highlight the work of individual disciplines, but they are not simply intended to recruit students to the university. Instead, we aim to return knowledge to the communities where real transformative work can be accomplished. Therefore, our work with youth could not be done alone because we lack the community expertise.

Central to the success of our program was our engagement of community partners with long-standing relationships with community educators, stakeholders, and leaders. We focused on partnering with community organizations with an education focus to their mission. Two of the three were youth education and enrichment organizations that provided activities such as summer programs, mentoring, tutoring, and college preparation. The third youth program was a summer internship program based at the university but partners with an inner-city high school. Each organization had personnel who interacted with the students regularly and outside our public health program. These contacts were key to our program as they were able to address the needs of the students, which included things such as transportation, housing, and behavioral issues.

## Innovative Integrative Curriculum

Our public health curriculum for our five half-day camp focused on the role of drones in public health. Through didactic lectures and discussions, students were first introduced to key concepts in public health each day, which included population health, prevention, emergency preparedness, and vaccination programs. Then, each day, there was a substantiative topic related to drones. Students learned how drones have been used to spray insecticide in malaria-endemic areas, how drones have been used to deliver blood products to remote areas, how drones collect air pollution data, and how drone-collected data can be analyzed. Topics were chosen to represent the foundational areas of public health, including epidemiology, health policy, environmental health sciences, and biostatistics. Discussions were not restricted to drone use in public health. On the contrary, students frequently inquired about topics related to the disease, population, or how society impacts the health condition. Interspersed in the public health material was time learning about drones.

Students learned to pilot palm-sized, first-person view (FPV) quadcopter drones. These drones weigh roughly 3 ounces and can reach a maximum speed of 10 m.p.h. in beginner mode. They are used for competitive drone racing, an emerging e-sport in which pilots can compete nationally. As a pilot, and consistent with STEM education, students also learned of the circuitry of the aircraft, the composition of its parts, and the role of the battery.

Throughout the week, students developed a case-based scenario in which they could use their drones in a public health setting. The exercise was to develop a drone-related intervention that could be used in a specific population to prevent a large-scale public health emergency. The resultant scenario was used as a basis for the drone race that concluded the week-long camp.

### Conclusion

We have successfully bridged an introduction to public health with drone racing, an amusing hands-on STEM-based activity. Students in our pilot program gained an appreciation for public health work, and nearly all of them would consider a career in public health. Our program is particularly impactful given the diverse students reached. We are preparing to scale-up our program and offer our curriculum to students in additional underserved communities.
